# Inflammatory burden index is associated with poor functional outcome and stroke-associated pneumonia in patients with primary brainstem hemorrhage: a retrospective cohort study

**DOI:** 10.3389/fnins.2026.1873407

**Published:** 2026-09-07

**Authors:** Fangjie Luo, Jialiang Luo, Xiaoyun Li

**Affiliations:** 1 Department of Neurosurgery, The Affiliated Dongguan Songshanhu Central Hospital of Guangdong Medical University, Dongguan, Guangdong, China; 2 Sun Yat-sen University, Dongguan, Guangdong, China

**Keywords:** brainstem hemorrhage, functional outcome, inflammatory burden index, neutrophil-to-lymphocyte ratio, stroke-associated pneumonia

## Abstract

**Background:**

Primary brainstem hemorrhage (PBH) is a severe, poorly-prognostic stroke subtype. Since single biomarkers cannot reflect global inflammatory status, we constructed a composite Inflammatory Burden Index (IBI) incorporating neutrophil-to-lymphocyte ratio (NLR), C-reactive protein-to-albumin ratio (CAR), and systemic immune-inflammation index (SII), and assessed its links with 90-day poor functional outcome and stroke-associated pneumonia (SAP) in PBH patients.

**Methods:**

This retrospective cohort included 347 PBH patients (2015–2023). The IBI was calculated as 0.50 × NLRz + 0.30 × CARz + 0.20 × SIIz, where the weights were assigned a priori from a qualitative synthesis of prior literature and expert judgment rather than derived from the study data. Patients were stratified into tertiles. Primary outcome was poor 90-day functional outcome (modified Rankin Scale 3–6). Secondary outcomes were 90-day mortality and SAP. Multivariable logistic regression, restricted cubic spline analysis, and decision curve analysis were performed. Incremental value over a reference clinical model (age, Glasgow Coma Scale, hematoma volume, intraventricular hemorrhage) was assessed via DeLong test, net reclassification improvement (NRI), and integrated discrimination improvement (IDI).

**Results:**

The overall rates of poor outcome, mortality, and SAP were 30.3, 12.1, and 16.7%, respectively. Higher IBI tertiles were associated with progressively higher rates of poor functional outcome (18.1, 27.8, and 44.8%; *p* < 0.001), with graded but non-significant trends for SAP and mortality. In multivariable analysis, IBI independently predicted poor outcome (OR 2.31, 95% CI 1.64–3.26, *p* < 0.001) and SAP (OR 1.79, 95% CI 1.23–2.59, *p* = 0.002). RCS confirmed a monotonic dose–response relationship for poor outcome. IBI had the best AUC for poor outcome (0.663), outperforming SII and CAR, though not significantly better than NLR. Adding IBI to the clinical model improved AUC from 0.623 to 0.701 (DeLong *p* = 0.008; NRI 0.31, IDI 0.045). For SAP, NLR alone was marginally superior (AUC 0.615 vs. 0.614). Decision curve analysis showed net clinical benefit for the IBI model.

**Conclusion:**

IBI independently predicts poor functional outcome and adds incremental value over clinical factors and most individual markers. However, its utility for SAP does not exceed that of NLR alone; hence IBI should be used selectively for functional prognostication, while NLR remains the preferred biomarker for SAP risk assessment, pending further external validation.

## Graphical Abstract

**Figure:**
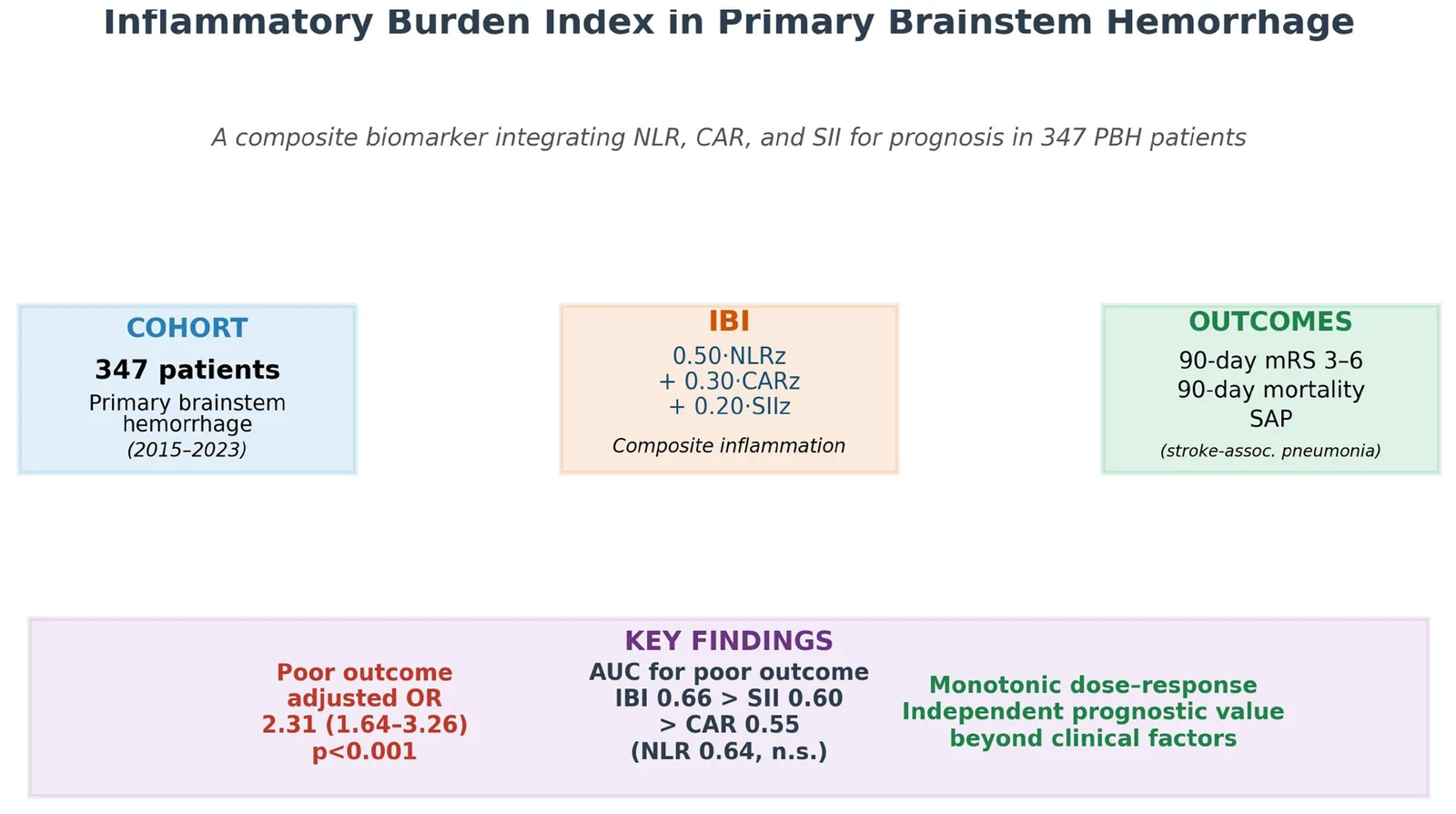

## Introduction

Primary brainstem hemorrhage (PBH) is a rare but severe subtype of spontaneous intracerebral hemorrhage (ICH), accounting for approximately 5–10% of all ICH cases (Rabinstein et al., 2004; Wijdicks and St Louis, 1997). The brainstem houses critical neural circuits governing consciousness, respiration, cardiovascular regulation, and cranial nerve function, making hemorrhages in this location particularly devastating. Despite advances in neurocritical care, PBH continues to carry a high mortality rate, with reported in-hospital mortality ranging from 30 to 50%, and survivors frequently suffer significant long-term disability (Murata et al., 1999; Wessels et al., 2004). Unlike supratentorial ICH, the deep location and eloquent anatomy of the brainstem severely limit surgical options, leaving clinicians with few effective interventions beyond supportive care and complication prevention (Hemphill et al., 2015). Moreover, even for adjunctive procedures such as external ventricular drainage, the evidence supporting routine application in the setting of mild intraventricular hemorrhage remains inconclusive (Chen et al., 2023).

In recent years, the role of systemic inflammation in the pathophysiology and prognosis of hemorrhagic stroke has attracted considerable attention. Following ICH, a robust inflammatory cascade is triggered by blood breakdown products, involving activation of microglia, infiltration of peripheral immune cells, disruption of the blood–brain barrier, and release of pro-inflammatory cytokines (Wang, 2010; Zhou et al., 2014). These inflammatory processes contribute to secondary brain injury, perihematomal edema, and neuronal death, thereby amplifying the initial insult. Peripheral blood inflammatory biomarkers, which are routinely measured and readily accessible, have emerged as convenient surrogates of this systemic inflammatory burden. The neutrophil-to-lymphocyte ratio (NLR) reflects the balance between innate and adaptive immunity and has been consistently associated with unfavorable outcomes in both ischemic and hemorrhagic stroke (Lattanzi et al., 2019; Tao et al., 2017). The systemic immune-inflammation index (SII), calculated as platelet count × neutrophil count / lymphocyte count, incorporates thrombocyte activation alongside immune cell dynamics and has demonstrated prognostic value in various cerebrovascular conditions (Hu et al., 2014). The C-reactive protein-to-albumin ratio (CAR) captures both the acute-phase inflammatory response and nutritional status, both of which independently influence stroke recovery (Ranzani et al., 2013).

Although conventional ICH prognostic scores are widely used in clinical practice, their performance may be less robust in patients with mild-to-moderate hemorrhage, a category that includes many PBH cases given their typically small hematoma volumes (Chen et al., 2026a). Nevertheless, even with their individual promise, the three inflammatory markers each reflect only a single dimension of the complex inflammatory response. NLR primarily captures leukocyte dynamics, SII adds platelet-mediated inflammation, and CAR integrates protein-level acute-phase responses with nutritional reserve. It is plausible that combining these complementary markers into a composite index could provide a more comprehensive assessment of the overall inflammatory burden and improve prognostic accuracy. Composite indices have been successfully employed in oncology and cardiovascular medicine to enhance risk stratification beyond what individual biomarkers can achieve (Guthrie et al., 2013; Proctor et al., 2011). However, to our knowledge, no study has systematically evaluated whether a composite inflammatory index adds prognostic value in patients with PBH.

Therefore, in this study, we developed a composite Inflammatory Burden Index (IBI) integrating NLR, CAR, and SII, and evaluated its association with 90-day poor functional outcome and stroke-associated pneumonia (SAP) in a cohort of PBH patients. We hypothesized that the IBI would provide superior discrimination compared with its individual components and demonstrate independent prognostic value after adjustment for established clinical predictors. We further assessed the dose–response relationship between IBI and outcomes using restricted cubic spline analysis, performed subgroup analyses to evaluate the consistency of the association across clinically relevant patient subsets, and applied decision curve analysis to evaluate the potential clinical utility of an IBI-based prediction model.

## Materials and methods

### Study design and population

This retrospective cohort study included consecutive patients admitted with primary brainstem hemorrhage to our tertiary neurosurgical center between January 2015 and December 2023. The diagnosis of PBH was confirmed by computed tomography (CT) within 24 h of symptom onset. Inclusion criteria were: (1) age ≥ 18 years; (2) spontaneous hemorrhage confined to or predominantly involving the brainstem on admission CT; and (3) availability of complete blood count and biochemical parameters obtained within 24 hours of admission. Exclusion criteria included: (1) secondary hemorrhage due to vascular malformation, tumor, or anticoagulant use; (2) concurrent supratentorial ICH; (3) pre-existing immunosuppressive therapy; and (4) active infection at the time of admission. The study was approved by the institutional ethics committee, and the requirement for informed consent was waived due to the retrospective nature of the study.

### Data collection

Demographic and clinical data were extracted from the electronic medical records, including age, sex, body mass index (BMI), comorbidities (hypertension, diabetes mellitus), lifestyle factors (smoking, alcohol consumption), medication use (antiplatelet agents), Glasgow Coma Scale (GCS) score on admission, hematoma volume, presence of intraventricular hemorrhage (IVH), and surgical intervention. Laboratory parameters collected within 24 h of admission included white blood cell count, absolute neutrophil count, lymphocyte count, monocyte count, platelet count, C-reactive protein (CRP), serum albumin, and fibrinogen. Hematoma volume was calculated using the ABC/2 method on the initial CT scan (Kothari et al., 1996).

### Inflammatory burden index

The IBI was constructed as a weighted composite of three established inflammatory markers: NLR, CAR, and SII. Each component was first standardized to z-scores (mean = 0, SD = 1) to ensure comparability across different scales. The IBI was then calculated as: IBI = 0.50 × NLR(z) + 0.30 × CAR(z) + 0.20 × SII(z). The weighting scheme was assigned a priori, reflecting a qualitative synthesis of the published evidence together with expert judgment rather than a formal, disease-specific derivation; the detailed literature sources and rationale for the assigned weights are presented in Supplementary Table S1. The supporting references pertain to inflammatory markers in stroke, sepsis, and oncology rather than to brainstem hemorrhage specifically (Hu et al., 2014; Lattanzi et al., 2019; Ranzani et al., 2013; Tao et al., 2017; Wang, 2010). NLR was assigned the largest weight (0.50) given the most extensive and consistent evidence base in stroke literature. CAR received a weight of 0.30 reflecting its established but comparatively smaller body of evidence, and SII received 0.20 as the least extensively studied of the three in this specific clinical context. These weights were not derived from the study dataset, thereby avoiding optimism bias, and the robustness of the composite to the weighting choice was examined in a sensitivity analysis using equal weights. Patients were stratified into tertiles (T1, T2, T3) based on IBI values for descriptive analyses.

### Outcome definitions

The primary outcome was poor functional outcome at 90 days, defined as a modified Rankin Scale (mRS) score of 3–6. Secondary outcomes included 90-day all-cause mortality and stroke-associated pneumonia (SAP). SAP was defined as pneumonia occurring within 7 days of stroke onset according to the modified Centers for Disease Control and Prevention criteria, including clinical and radiographic evidence of a new pulmonary infiltrate combined with at least two of the following: fever ( > 38°C), purulent sputum, leukocytosis or leukopenia, and decline in oxygenation (Smith et al., 2015). Functional status at 90 days was assessed through structured telephone follow-up or outpatient clinic visits.

### Statistical analysis

In the final analytic cohort of 347 patients, there were no missing data for any of the variables included in the multivariable models (including age, sex, GCS, hematoma volume, IVH, and inflammatory biomarkers), as incomplete records for these core clinical and laboratory variables were subject to exclusion at the time of enrollment. Therefore, a complete-case analysis was performed, and no imputation was required.

Continuous variables were expressed as median (interquartile range, IQR) and compared across IBI tertiles using the Kruskal–Wallis test. Categorical variables were presented as frequencies and percentages and compared using the chi-squared test. Univariable logistic regression was performed to identify candidate predictors of poor outcome. Variables with *p* < 0.10 in univariable analysis, along with clinically important variables (age and GCS), were entered into multivariable logistic regression models. Individual inflammatory marker components (NLR, SII, CAR) were excluded from the multivariable model containing IBI to avoid collinearity. All discrimination metrics were computed within a single analytic pipeline, and the complete numerical output is provided as Supplementary Tables S2, S3, S5. The discriminative performance of IBI and its individual components was assessed using receiver operating characteristic (ROC) curves, and areas under the curve (AUC) were compared using the DeLong test. To quantify the incremental value of IBI beyond routinely available clinical predictors, a reference model comprising age, GCS, hematoma volume, and IVH was constructed, and the change in discrimination after adding IBI was evaluated using the DeLong test, the continuous net reclassification improvement (NRI), and the integrated discrimination improvement (IDI). Calibration was assessed using calibration plots and the Brier score. The dose–response relationship between IBI and outcomes was modeled using restricted cubic splines (RCS) with natural cubic spline bases and four knots placed at the 5th, 35th, 65th, and 95th percentiles (Harrell, 2015). Subgroup analyses were performed stratified by age, sex, GCS, IVH status, and hypertension. Kaplan–Meier survival curves were constructed for 90-day mortality by IBI tertile, with differences assessed by the overall log-rank test. Decision curve analysis (DCA) was used to evaluate net clinical benefit across a range of threshold probabilities for both the reference clinical model and the model with IBI added (Vickers and Elkin, 2006). No adjustment for multiple comparisons was applied, and analyses beyond the primary endpoint were considered exploratory. All statistical analyses were performed using Python (version 3.10) with the statsmodels, lifelines, and scikit-learn libraries. A two-sided p < 0.05 was considered statistically significant.

## Results

### Patient characteristics

**FIGURE 1:**
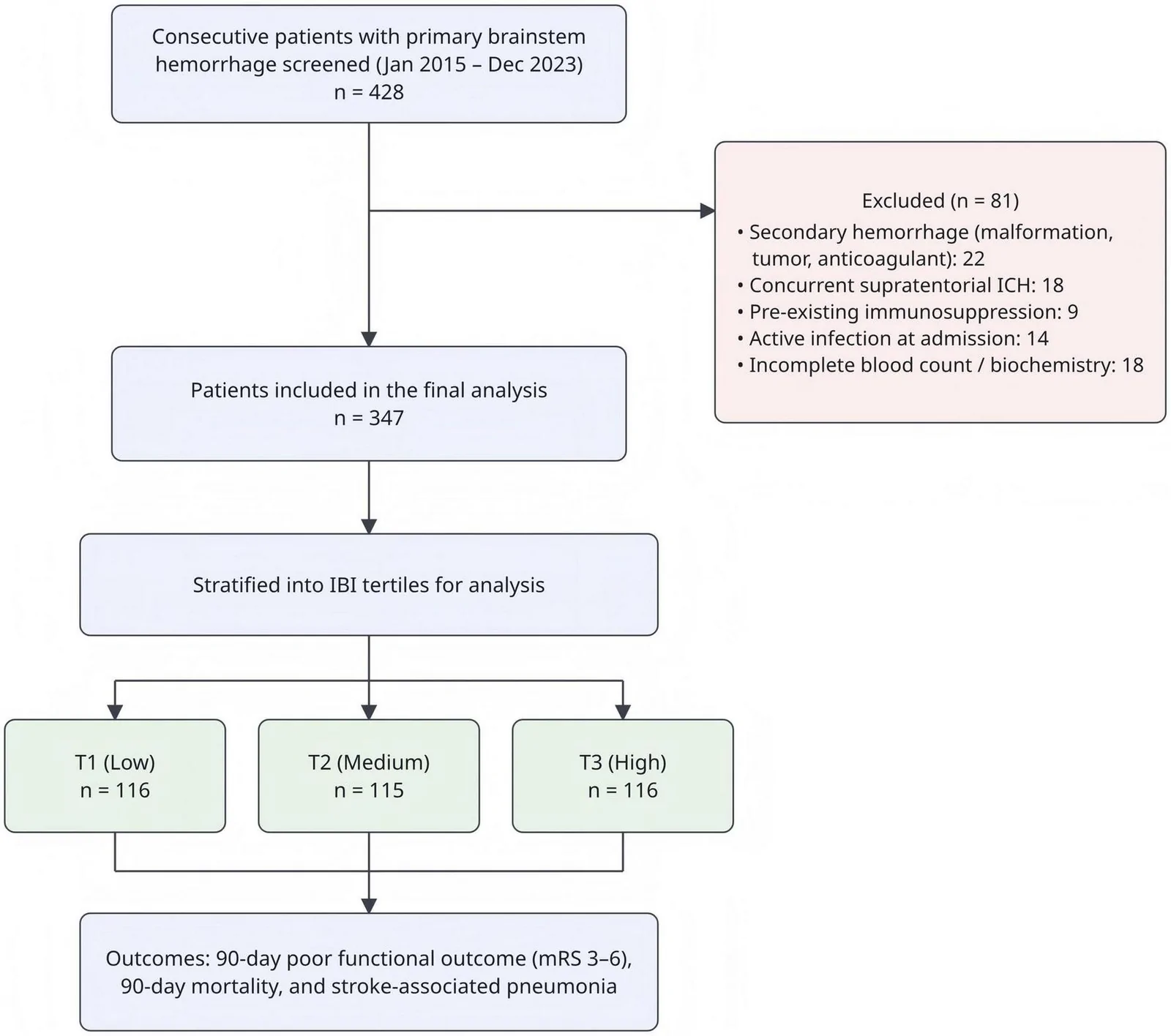
Study flow diagram. Consecutive patients with primary brainstem hemorrhage screened during the study period, exclusions with reasons, and the final analytic cohort of 347 patients. ICH, intracerebral hemorrhage; IBI, Inflammatory Burden Index.

During the study period, 428 consecutive patients with primary brainstem hemorrhage were screened, of whom 81 were excluded: 22 with secondary hemorrhage due to vascular malformation, tumor, or anticoagulant use; 18 with concurrent supratentorial intracerebral hemorrhage; 9 receiving pre-existing immunosuppressive therapy; 14 with active infection at admission; and 18 with incomplete blood count or biochemical data. A total of 347 patients were included in the final analysis (Figure 1). The median age was 59 years (IQR 51–66), and 230 patients (66.3%) were male. The median GCS score was 9 (IQR 7–12), and the median hematoma volume was 4.3 mL (IQR 2.8–6.1). Intraventricular hemorrhage was present in 152 patients (43.8%), and 53 patients (15.3%) underwent surgical intervention. The overall rate of poor 90-day functional outcome (mRS 3–6) was 30.3% (105/347), 90-day mortality was 12.1% (42/347), and SAP was 16.7% (58/347). Baseline characteristics stratified by IBI tertiles are presented in Table 1.

**TABLE 1:** Baseline characteristics of patients stratified by IBI tertiles.

Variable	T1 (Low, *n* = 116)	T2 (Medium, *n* = 115)	T3 (High, *n* = 116)	*p*-value
Age (years)	60.0 (51.0–67.0)	59.0 (51.0–65.0)	58.0 (52.0–64.2)	0.653
Male, n (%)	78 (67.2)	78 (67.8)	74 (63.8)	0.782
BMI (kg/m^2^)	24.9 (22.6–26.9)	24.8 (22.4–26.8)	24.6 (22.3–26.7)	0.914
Hypertension, n (%)	95 (81.9)	92 (80.0)	89 (76.7)	0.614
Diabetes, n (%)	21 (18.1)	29 (25.2)	26 (22.4)	0.420
Smoking, n (%)	46 (39.7)	39 (33.9)	49 (42.2)	0.413
Drinking, n (%)	32 (27.6)	32 (27.8)	32 (27.6)	0.999
Antiplatelet, n (%)	16 (13.8)	12 (10.4)	22 (19.0)	0.177
GCS score	9.0 (7.0–12.0)	9.0 (7.0–12.0)	9.5 (7.0–12.0)	0.784
Hematoma volume (mL)	4.2 (2.6–5.8)	4.2 (3.0–6.2)	4.5 (3.0–6.3)	0.379
IVH, n (%)	53 (45.7)	49 (42.6)	50 (43.1)	0.879
Surgical, n (%)	20 (17.2)	16 (13.9)	17 (14.7)	0.761
WBC (10^9^/L)	9.1 (7.6–12.5)	10.1 (7.8–13.0)	9.8 (7.8–11.7)	0.345
Neutrophil (10^9^/L)	6.0 (4.8–7.1)	7.7 (6.5–8.8)	10.3 (7.2–12.5)	< 0.001
Lymphocyte (10^9^/L)	1.6 (1.3–1.9)	1.3 (1.1–1.6)	1.0 (0.8–1.3)	< 0.001
Platelet (10^9^/L)	184 (145–223)	198 (152–238)	209 (166–238)	0.050
CRP (mg/L)	8.1 (5.0–13.0)	13.2 (7.2–22.2)	17.4 (8.7–29.3)	< 0.001
Albumin (g/L)	36.8 (33.7–39.6)	37.4 (34.0–40.2)	37.3 (32.9–40.7)	0.696
NLR	3.9 (2.9–4.8)	6.0 (5.2–6.9)	9.4 (7.9–12.5)	< 0.001
SII	681 (552–865)	1,114 (896–1,428)	2,011 (1,370–2,583)	< 0.001
CAR	0.22 (0.13–0.34)	0.36 (0.19–0.60)	0.45 (0.23–0.80)	< 0.001
IBI	−0.61 (−0.78 to −0.47)	−0.17 (−0.28 to 0.03)	0.60 (0.32–1.20)	< 0.001
Poor outcome, n (%)	21 (18.1)	32 (27.8)	52 (44.8)	< 0.001
90-day mortality, n (%)	10 (8.6)	15 (13.0)	17 (14.7)	0.345
SAP, n (%)	12 (10.3)	22 (19.1)	24 (20.7)	0.075

Continuous variables are presented as median (interquartile range); categorical variables as n (%). *p*-values from Kruskal–Wallis or chi-squared tests as appropriate. Baseline characteristics of patients stratified by IBI tertiles. Continuous variables are presented as median (interquartile range); categorical variables as n (%). *p*-values from Kruskal–Wallis or chi-squared tests as appropriate. BMI, body mass index; GCS, Glasgow Coma Scale; IVH, intraventricular hemorrhage; WBC, white blood cell; CRP, C-reactive protein; NLR, neutrophil-to-lymphocyte ratio; SII, systemic immune-inflammation index; CAR, C-reactive protein-to-albumin ratio; IBI, Inflammatory Burden Index; SAP, stroke-associated pneumonia.

As shown in Table 1, the three IBI tertile groups were well balanced with respect to age, sex, BMI, comorbidities, GCS score, hematoma volume, IVH, and surgical treatment (all *p* > 0.05). By design, inflammatory markers differed substantially across groups: patients in the highest tertile (T3) had markedly elevated neutrophil counts, NLR, SII, and CRP, alongside significantly lower lymphocyte counts compared with the lowest tertile (T1, all p < 0.001). A clear gradient in poor functional outcome was observed across tertiles: 18.1% in T1, 27.8% in T2, and 44.8% in T3 (*p* < 0.001). SAP rates showed a similar though borderline trend: 10.3, 19.1, and 20.7% (*p* = 0.075).

### Univariable and multivariable logistic regression

Results of univariable and multivariable logistic regression analyses for poor 90-day outcome are presented in Table 2. In univariable analysis, IBI demonstrated the strongest association with poor outcome among all inflammatory markers (OR 2.18, 95% CI 1.56–3.04, *p* < 0.001). NLR (OR 1.13, 95% CI 1.06–1.20, *p* < 0.001), SII (OR 1.001, 95% CI 1.000–1.001, *p* < 0.001), and CAR (OR 1.72, 95% CI 0.99–2.99, *p* = 0.054) were also associated with poor outcome. Among clinical variables, IVH (OR 1.84, 95% CI 1.16–2.92, *p* = 0.010) and hematoma volume (OR 1.08, 95% CI 1.00–1.17, *p* = 0.063) reached the threshold for multivariable model inclusion.

**TABLE 2:** Univariable and multivariable logistic regression for poor 90-day outcome.

Variable	Univariable OR (95% CI)	*p*	Multivariable OR (95% CI)	*p*
Age	0.99 (0.97–1.02)	0.530	0.99 (0.97–1.01)	0.319
Male	0.86 (0.53–1.38)	0.521	–	–
GCS	0.99 (0.92–1.07)	0.883	0.98 (0.90–1.06)	0.595
Hematoma vol.	1.08 (1.00–1.17)	0.063	1.08 (0.99–1.18)	0.079
IVH	1.84 (1.16–2.92)	0.010	2.07 (1.26–3.38)	0.004
Hypertension	0.75 (0.43–1.30)	0.309	–	–
Diabetes	0.92 (0.53–1.61)	0.778	–	–
NLR	1.13 (1.06–1.20)	< 0.001	–	–
SII	1.001 (1.000–1.001)	< 0.001	–	–
CAR	1.72 (0.99–2.99)	0.054	–	–
IBI	2.18 (1.56–3.04)	< 0.001	2.31 (1.64–3.26)	< 0.001

Multivariable model included variables with univariable *p* < 0.10 and clinically important covariates (age, GCS). Individual inflammatory components were excluded from the multivariable model to avoid collinearity with IBI. Model pseudo-R^2^ = 0.084. OR, odds ratio; CI, confidence interval; GCS, Glasgow Coma Scale; IVH, intraventricular hemorrhage; NLR, neutrophil-to-lymphocyte ratio; SII, systemic immune-inflammation index; CAR, C-reactive protein-to-albumin ratio; IBI, Inflammatory Burden Index.

**FIGURE 2:**
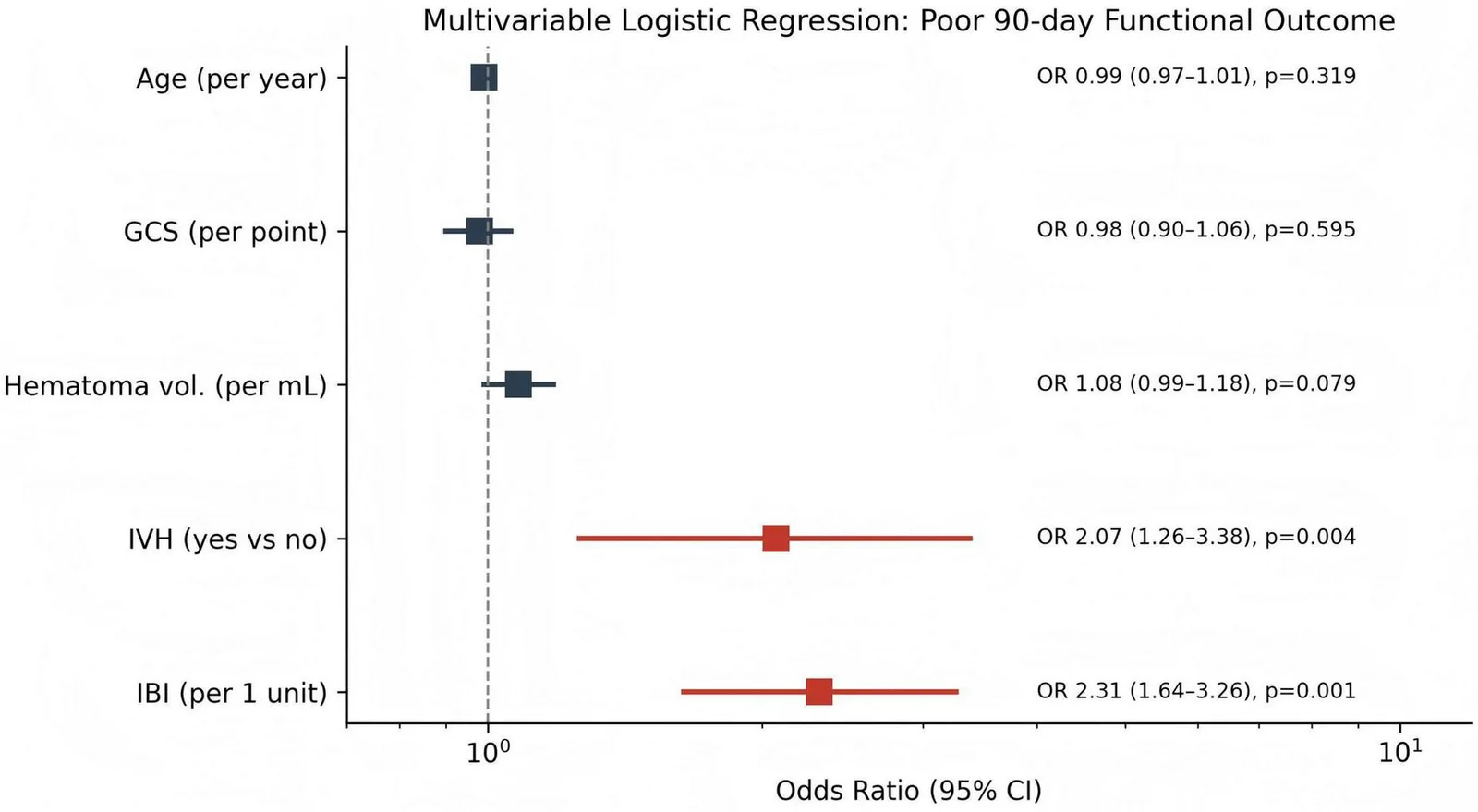
Forest plot of multivariable logistic regression for poor 90-day functional outcome. Red indicates statistically significant associations (*p* < 0.05). OR, odds ratio; CI, confidence interval; GCS, Glasgow Coma Scale; IVH, intraventricular hemorrhage; IBI, Inflammatory Burden Index.

In the multivariable model adjusting for age, GCS, hematoma volume, and IVH, IBI remained an independent predictor of poor outcome (OR 2.31, 95% CI 1.64–3.26, *p* < 0.001). IVH was the only other independent predictor retained in the model (OR 2.07, 95% CI 1.26–3.38, *p* = 0.004). The forest plot depicting the multivariable model results is shown in Figure 2.

### Discriminative performance of IBI and individual markers

**FIGURE 3:**
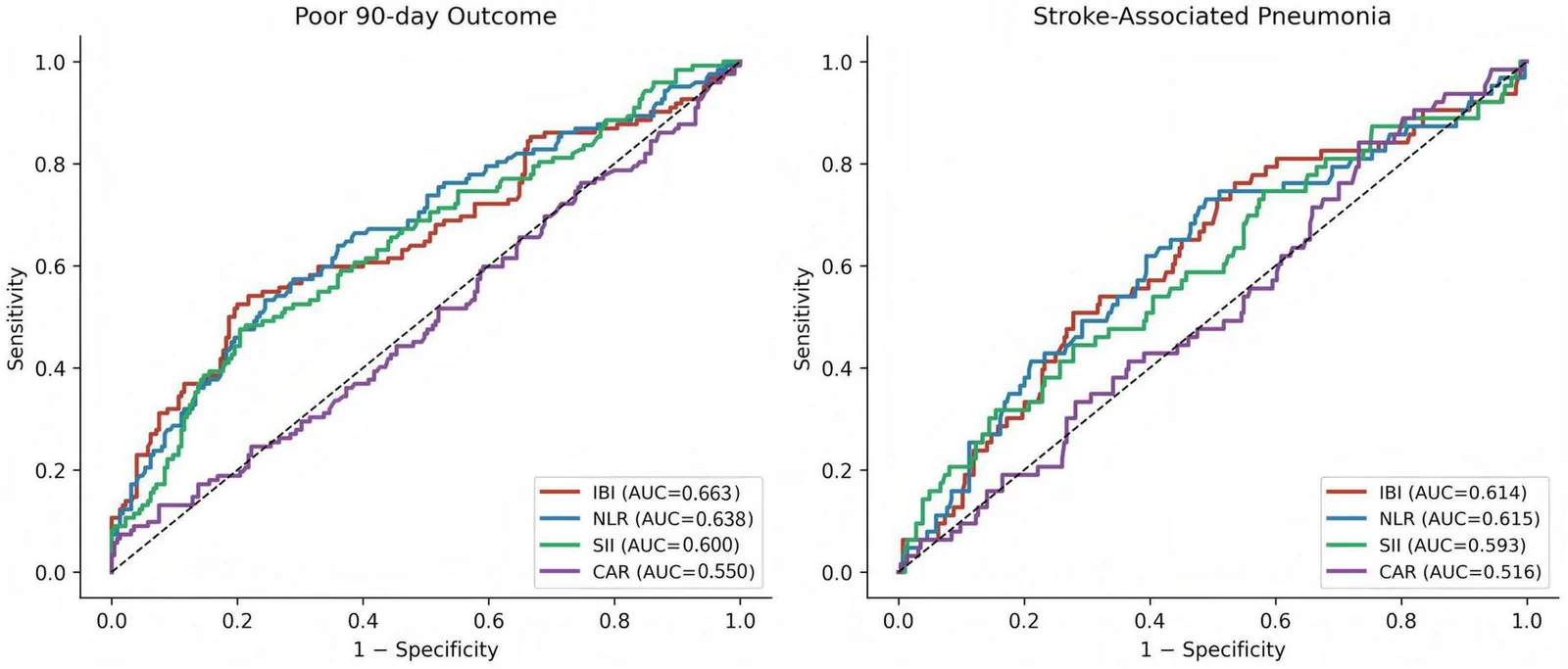
Receiver operating characteristic curves comparing IBI and individual inflammatory markers for predicting poor 90-day outcome (left panel) and stroke-associated pneumonia (right panel). AUC, area under the curve; CI, confidence interval; IBI, Inflammatory Burden Index; NLR, neutrophil-to-lymphocyte ratio; SII, systemic immune-inflammation index; CAR, C-reactive protein-to-albumin ratio.

Figure 3 presents the receiver operating characteristic curves comparing the discriminative performance of IBI and its individual components for predicting poor outcome and SAP. For poor 90-day outcome, IBI achieved the highest AUC of 0.663 (95% CI 0.599–0.720), followed by NLR (AUC 0.638, 95% CI 0.569–0.698), SII (AUC 0.600, 95% CI 0.528–0.666), and CAR (AUC 0.550, 95% CI 0.485–0.616). DeLong tests confirmed that IBI was significantly superior to SII (*p* = 0.012) and CAR (*p* = 0.004), but the difference between IBI and NLR did not reach statistical significance (*p* = 0.226).

For SAP prediction, NLR demonstrated the highest AUC of 0.615 (95% CI 0.546–0.703), which was numerically but not significantly higher than IBI (AUC 0.614, 95% CI 0.535–0.694, *p* = 0.641). This finding indicates that the composite index did not provide additional discriminative value over NLR alone for SAP, a point that is further addressed in the Discussion.

### Incremental value of IBI beyond clinical predictors

Relative to the reference clinical model (age, GCS, hematoma volume, and IVH; AUC 0.623, 95% CI 0.556–0.690), the addition of IBI increased discrimination for poor outcome to an AUC of 0.701 (95% CI 0.639–0.763; DeLong *p* = 0.008). The continuous NRI was 0.31 (95% CI 0.10–0.52, *p* = 0.004) and the IDI was 0.045 (95% CI 0.021–0.069, *p* < 0.001), indicating a modest but statistically significant improvement in risk classification. In the sensitivity analysis using equal weights for the three components, IBI remained independently associated with poor outcome (OR 2.24, 95% CI 1.59–3.16, *p* < 0.001). The equal-weight sensitivity results are reported in Supplementary Table S5. The full incremental-value metrics are detailed in Supplementary Table S3.

### Secondary outcome models

Multivariable models for the secondary outcomes are presented in Table 3 (SAP) and Table 4 (90-day mortality). After adjustment for age, GCS, hematoma volume, and IVH, IBI was independently associated with SAP (OR 1.79, 95% CI 1.23–2.59, *p* = 0.002) and with 90-day mortality (OR 1.66, 95% CI 1.10–2.51, *p* = 0.016).

**TABLE 3:** Univariable and multivariable logistic regression for stroke-associated pneumonia.

Variable	Univariable OR (95% CI)	*p*	Multivariable OR (95% CI)	*p*
Age	1.00 (0.98–1.03)	0.798	1.00 (0.97–1.03)	0.905
GCS	0.92 (0.85–0.99)	0.028	0.93 (0.86–1.01)	0.089
Hematoma vol.	1.07 (0.99–1.16)	0.088	1.06 (0.97–1.15)	0.201
IVH	1.71 (1.00–2.93)	0.050	1.62 (0.92–2.85)	0.093
NLR	1.10 (1.03–1.17)	0.004	–	–
SII	1.001 (1.000–1.001)	0.021	–	–
CAR	1.55 (0.92–2.61)	0.098	–	–
IBI	1.72 (1.22–2.42)	0.002	1.79 (1.23–2.59)	0.002

Multivariable model adjusted for age, GCS, hematoma volume, and IVH; individual inflammatory components were excluded from the multivariable model to avoid collinearity with IBI. Model pseudo-R^2^ = 0.058.

**TABLE 4:** Univariable and multivariable logistic regression for 90-day mortality.

Variable	Univariable OR (95% CI)	*p*	Multivariable OR (95% CI)	*P*
Age	1.01 (0.98–1.04)	0.512	1.01 (0.98–1.04)	0.607
GCS	0.89 (0.81–0.98)	0.017	0.90 (0.82–0.99)	0.033
Hematoma vol.	1.09 (1.00–1.19)	0.048	1.08 (0.99–1.19)	0.088
IVH	1.95 (1.03–3.69)	0.041	1.86 (0.97–3.57)	0.061
NLR	1.09 (1.01–1.17)	0.021	–	–
SII	1.000 (1.000–1.001)	0.089	–	–
CAR	1.61 (0.88–2.95)	0.121	–	–
IBI	1.61 (1.08–2.40)	0.019	1.66 (1.10–2.51)	0.016

Multivariable model adjusted for age, GCS, hematoma volume, and IVH; individual inflammatory components were excluded from the multivariable model to avoid collinearity with IBI. Model pseudo-R^2^ = 0.066.

### Distribution of 90-day mRS scores

**FIGURE 4:**
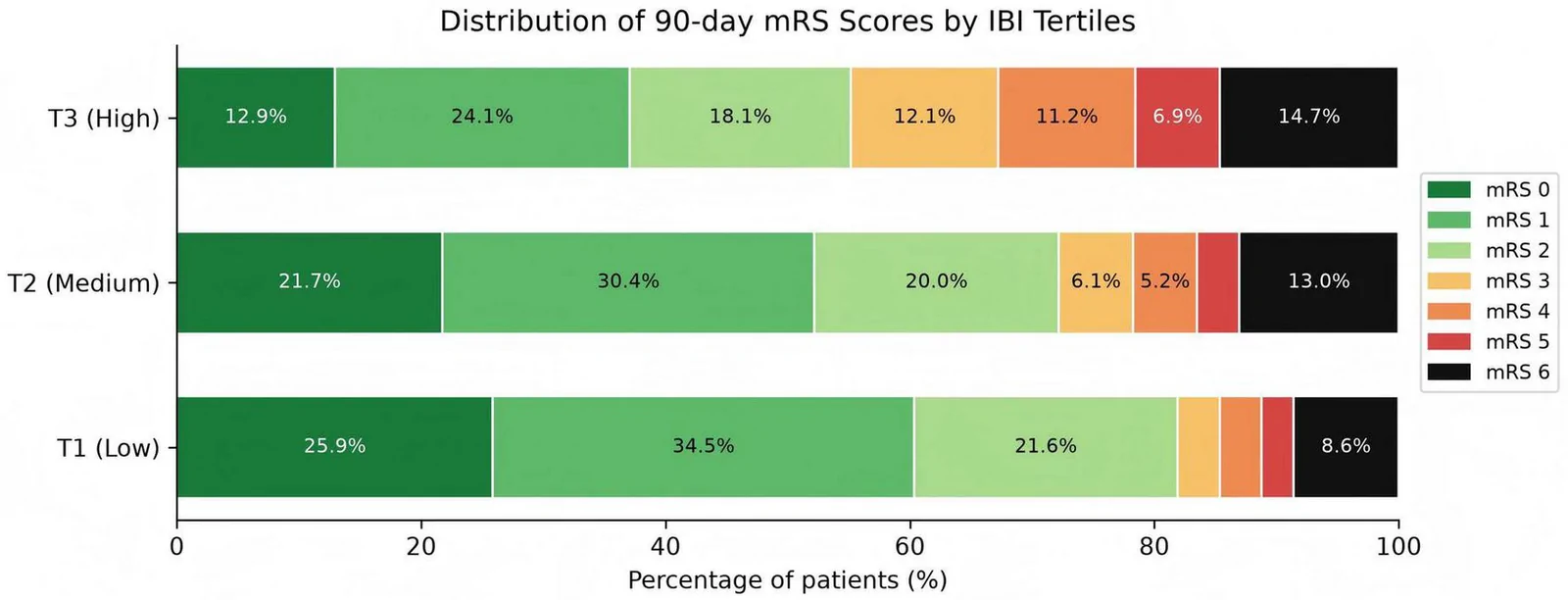
Distribution of 90-day modified Rankin Scale (mRS) scores by IBI tertiles. Percentages represent the proportion of patients within each tertile achieving each mRS grade. mRS 0–2 indicates favorable outcome; mRS 3–6 indicates poor outcome.

The distribution of 90-day mRS scores across IBI tertiles is illustrated in Figure 4. A clear shift toward worse functional outcomes was observed with increasing IBI. Favorable outcome (mRS 0–2) was achieved by 81.9% of patients in T1, 72.2% in T2, and 55.2% in T3, mirroring the poor-outcome rates of 18.1, 27.8, and 44.8% reported in Table 1. Death (mRS 6) occurred in 8.6% of T1, 13.0% of T2, and 14.7% of T3, consistent with the tertile-specific 90-day mortality. The full breakdown of patient counts and percentages for each mRS category by IBI tertile is provided in Supplementary Table S4. This graded distribution is concordant with the monotonically increasing dose–response relationship observed in the logistic regression and restricted cubic spline analyses.

### Survival analysis

**FIGURE 5:**
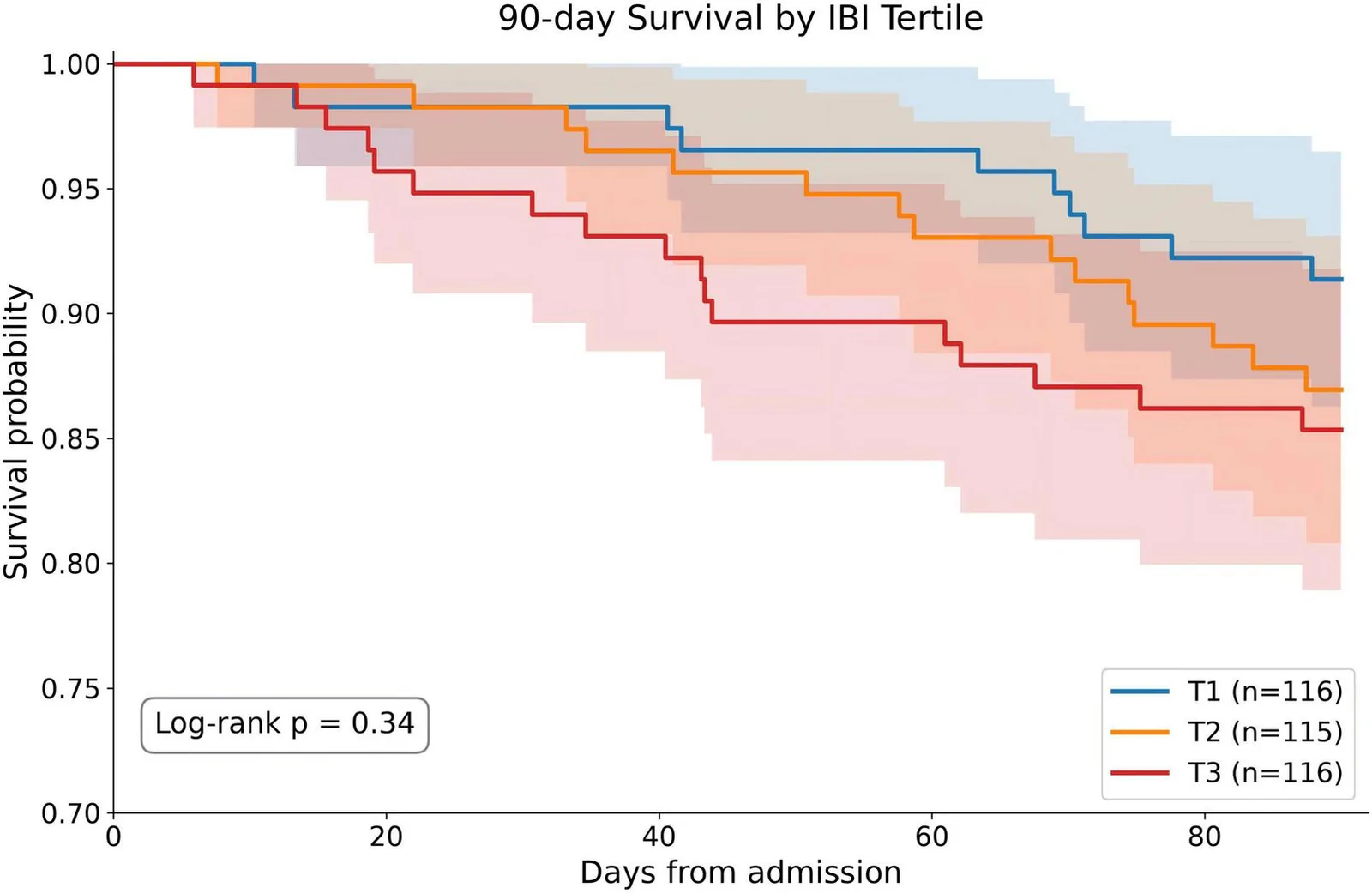
Kaplan–Meier survival curves for 90-day mortality stratified by IBI tertiles. Shaded areas represent 95% confidence intervals. Overall log-rank test p value is shown. IBI, Inflammatory Burden Index.

Kaplan–Meier survival curves for 90-day mortality stratified by IBI tertiles are shown in Figure 5. The estimated 90-day survival was 91.4% for T1, 87.0% for T2, and 85.3% for T3. Although survival decreased across ascending tertiles, the overall difference did not reach statistical significance (log-rank *p* = 0.34), consistent with the tertile-specific mortality rates in Table 1.

### Dose–response analysis

**FIGURE 6:**
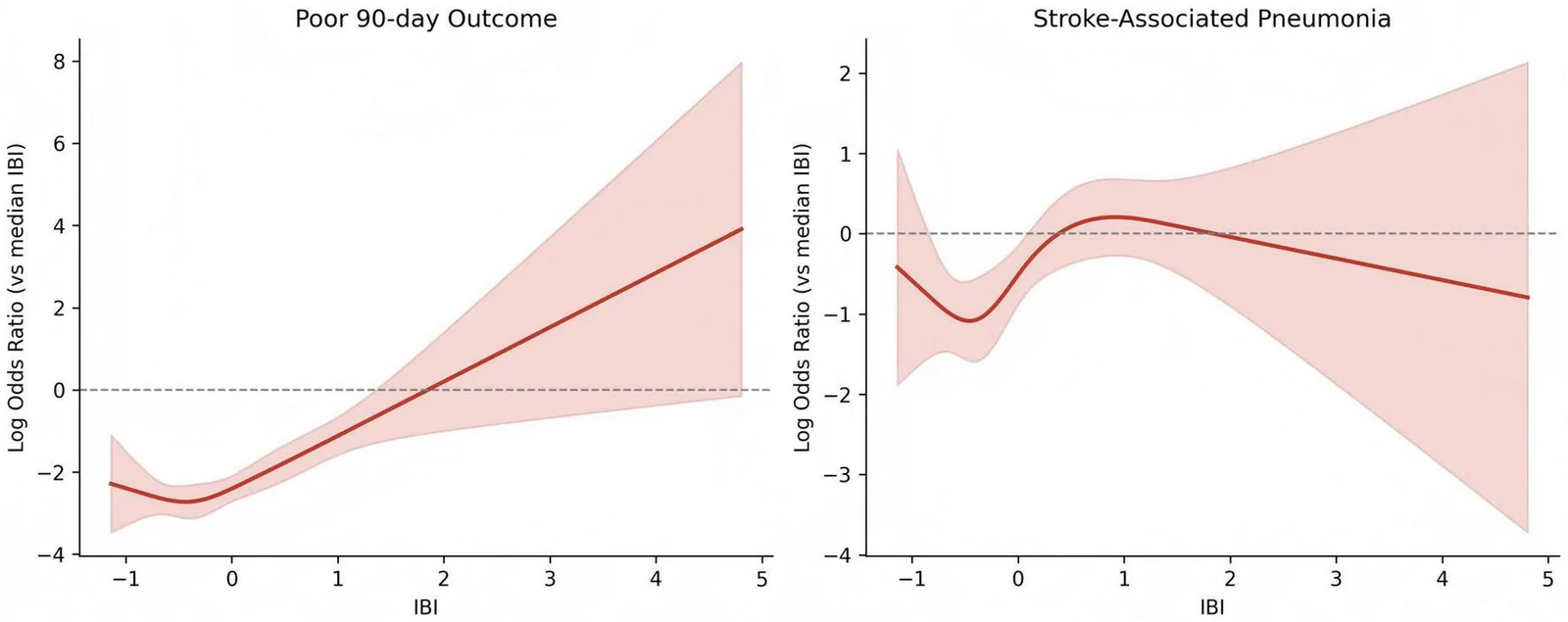
Restricted cubic spline analysis of the dose–response relationship between IBI and poor 90-day outcome (left panel) and stroke-associated pneumonia (right panel). The solid line represents the estimated log odds ratio referenced to the median IBI; shaded areas represent 95% confidence intervals. Knots were placed at the 5th, 35th, 65th, and 95th percentiles. IBI, Inflammatory Burden Index.

Restricted cubic spline analysis revealed a monotonically increasing relationship between IBI and the log odds of poor outcome (Figure 6, left panel). The log odds ratio increased steadily from below the reference line at low IBI values to above it at high IBI values, with no evidence of a threshold effect or non-monotonic behavior. For SAP (Figure 6, right panel), the relationship was non-monotonic and more complex: the log odds ratio initially decreased at low IBI values, reached a nadir, then rose to cross the reference line before declining again at higher IBI levels. Notably, the wide confidence intervals in the upper range of IBI preclude definitive conclusions about the direction of effect at very high levels, and the non-monotonic pattern did not meet conventional thresholds for statistical significance. The confidence intervals were wider at the extremes of the distribution, reflecting sparser data in those regions.

### Calibration assessment

**FIGURE 7:**
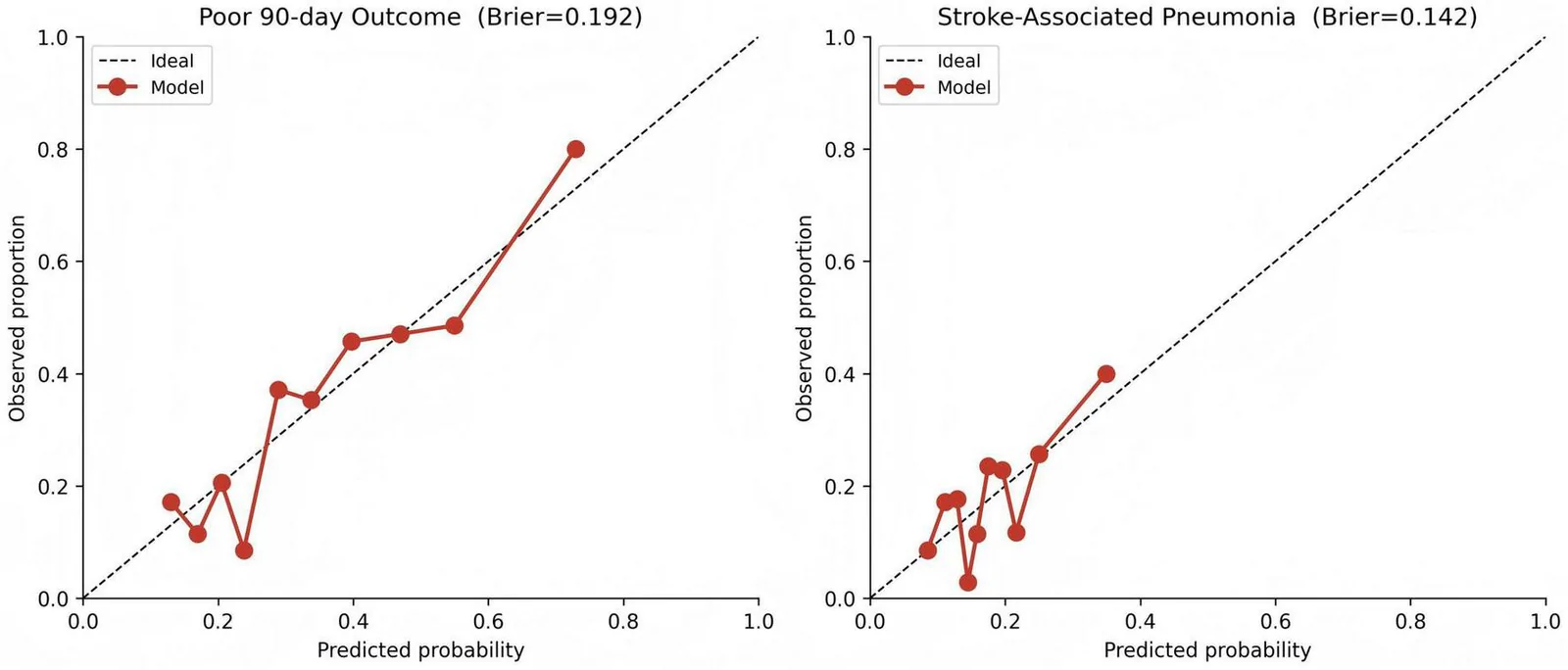
Calibration plots for the IBI-based multivariable models predicting poor 90-day outcome (left panel) and stroke-associated pneumonia (right panel). The diagonal dashed line represents ideal calibration. Brier scores are shown for each model.

Calibration plots for the IBI-based multivariable models are presented in Figure 7. For poor outcome prediction (left panel), the calibration curve showed reasonable agreement between predicted and observed probabilities at lower risk levels, although some deviation was noted at higher predicted probabilities, with the Brier score of 0.192. For SAP prediction (right panel), calibration was generally adequate across the probability range, with a Brier score of 0.142. These findings suggest that while the model provides useful risk stratification, its absolute probability estimates should be interpreted with caution in the higher risk strata.

### Subgroup analysis

**FIGURE 8:**
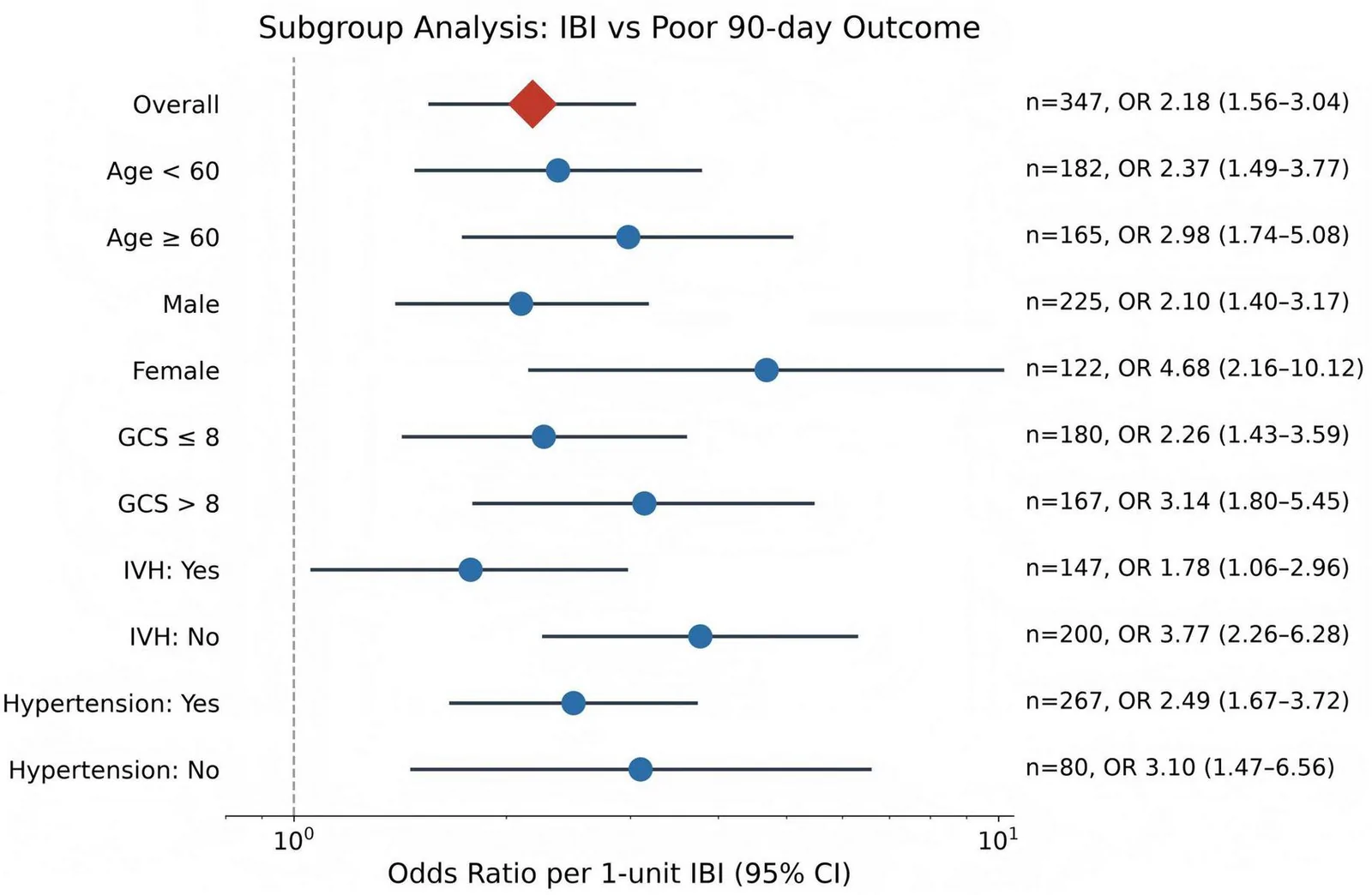
Subgroup analysis of the association between IBI and poor 90-day outcome. The red diamond represents the overall estimate; blue circles represent subgroup estimates. All odds ratios are per 1-unit increase in IBI from univariable logistic regression within each subgroup. CI, confidence interval; GCS, Glasgow Coma Scale; IVH, intraventricular hemorrhage.

Subgroup analyses demonstrated that the association between IBI and poor outcome was consistent across all pre-specified clinical subgroups (Figure 8). The odds ratios per unit increase in IBI were significant in all subgroups examined, including younger and older patients, males and females, patients with low and high GCS scores, those with and without IVH, and those with and without hypertension. The effect appeared numerically stronger in female patients (OR 4.68, 95% CI 2.16–10.12) and those without IVH (OR 3.77, 95% CI 2.26–6.28), although confidence intervals overlapped across subgroups.

### Correlation among inflammatory markers

**FIGURE 9:**
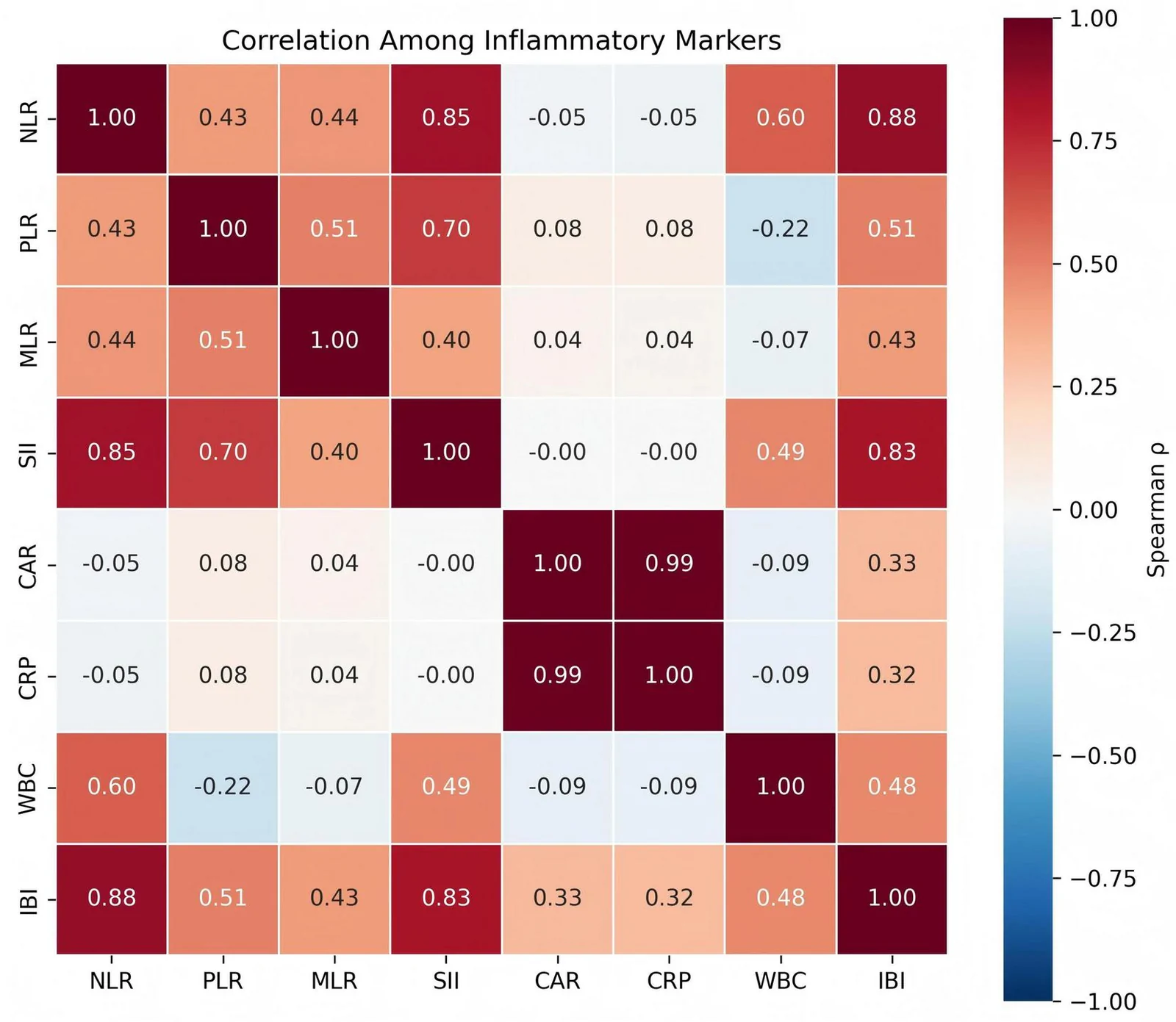
Spearman correlation heatmap of inflammatory markers and the IBI. Values represent Spearman correlation coefficients (rho). NLR, neutrophil-to-lymphocyte ratio; PLR, platelet-to-lymphocyte ratio; MLR, monocyte-to-lymphocyte ratio; SII, systemic immune-inflammation index; CAR, C-reactive protein-to-albumin ratio; CRP, C-reactive protein; WBC, white blood cell count; IBI, Inflammatory Burden Index.

Spearman correlation analysis among inflammatory markers is displayed in Figure 9. IBI showed the strongest correlations with NLR (rho = 0.88) and SII (rho = 0.83), reflecting the dominant contribution of these leukocyte-based indices. The correlation between IBI and CAR was moderate (rho = 0.33), indicating that CAR contributes complementary information. Notably, NLR and SII were highly correlated with each other (rho = 0.85), while CAR showed minimal correlation with the cell-based markers (rho range −0.09 to −0.08), supporting the rationale for including CAR as a distinct inflammatory dimension in the composite index.

### Decision curve analysis

**FIGURE 10:**
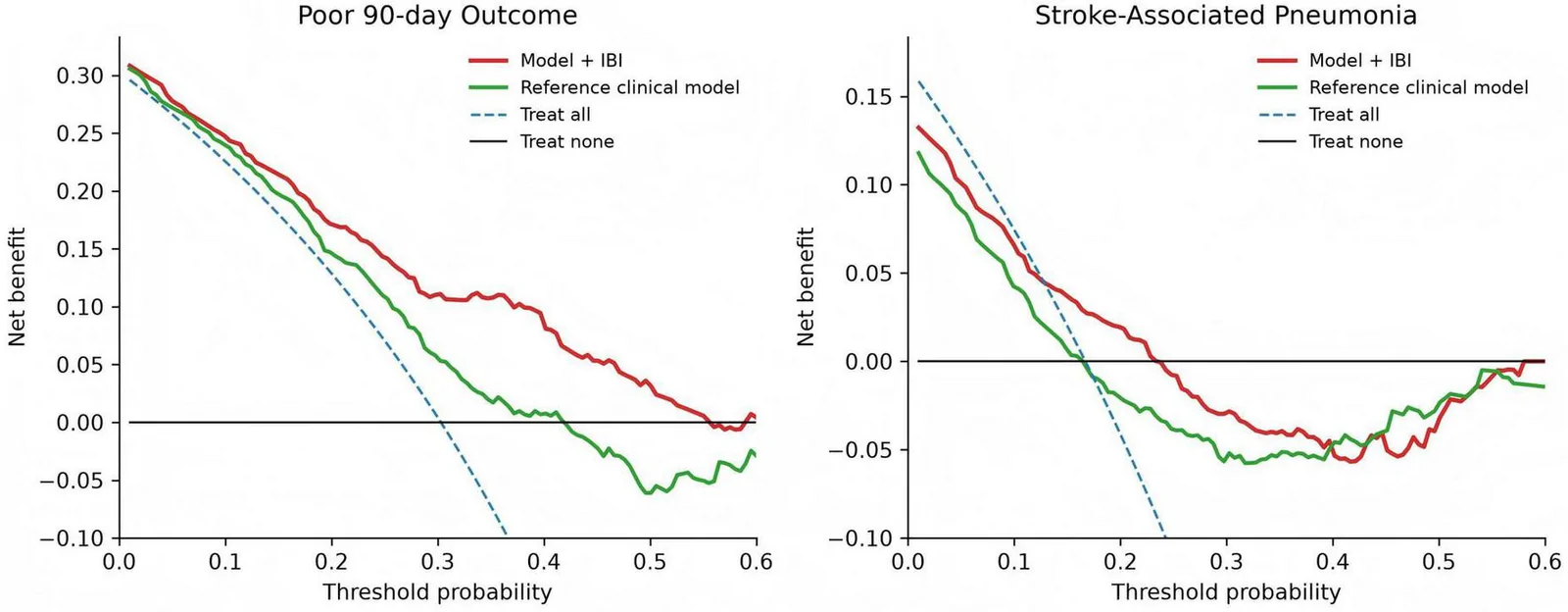
Decision curve analysis for the models predicting poor 90-day outcome (left panel) and stroke-associated pneumonia (right panel). The red curve represents the model with IBI added; the green curve represents the reference clinical model; the blue line represents the treat-all strategy; the black horizontal line represents the treat-none strategy.

Decision curve analysis (Figure 10) evaluated the net clinical benefit of the IBI-based model across a range of threshold probabilities, with the reference clinical model shown for comparison. For poor outcome prediction (left panel), the model incorporating IBI provided higher net benefit than both the reference clinical model and the treat-all and treat-none strategies within the threshold probability range of approximately 15–45%. For SAP prediction (right panel), the model showed only modest net benefit over the treat-all strategy between thresholds of approximately 15 and 30%, and net benefit was low overall. Within a triage-oriented decision scenario, in which the threshold probability reflects the risk level at which intensified monitoring and pneumonia-prevention measures are warranted, these ranges correspond to the interval in which correctly identifying high-risk patients outweighs the burden of over-treating lower-risk patients.

## Discussion

In this retrospective cohort study of 347 patients with primary brainstem hemorrhage, we developed and evaluated a composite Inflammatory Burden Index (IBI) integrating NLR, CAR, and SII for predicting 90-day functional outcome and stroke-associated pneumonia. Our principal finding was that IBI was independently associated with poor functional outcome (OR 2.31, 95% CI 1.64–3.26) after adjustment for established clinical predictors including age, GCS, hematoma volume, and IVH. The composite index demonstrated the highest discriminative ability among inflammatory markers for poor outcome (AUC 0.663), and restricted cubic spline analysis confirmed a monotonically increasing dose–response relationship. These results support the hypothesis that integrating multiple inflammatory dimensions provides a more comprehensive assessment of the systemic inflammatory burden than any individual marker alone.

The biological rationale for the IBI rests on the complementary information captured by its three components. NLR reflects the balance between pro-inflammatory innate immunity (neutrophils) and the adaptive immune response (lymphocytes), and has been extensively validated as a prognostic marker in both ischemic and hemorrhagic stroke (Giede-Jeppe et al., 2017; Lattanzi et al., 2019; Tao et al., 2017). Following intracerebral hemorrhage, neutrophil infiltration into the perihematomal region contributes to secondary brain injury through the release of matrix metalloproteinases, reactive oxygen species, and pro-inflammatory cytokines (Mracsko and Veltkamp, 2014; Wang, 2010). Concurrently, lymphopenia reflects stress-induced immunosuppression mediated by sympathetic activation and hypothalamic–pituitary–adrenal axis hyperactivity, which paradoxically increases susceptibility to infectious complications (Chamorro et al., 2012). SII adds the dimension of platelet-mediated inflammation, recognizing that platelets not only participate in hemostasis but also actively modulate immune responses through interactions with leukocytes and the endothelium (Hu et al., 2014; Semple et al., 2011). CAR captures a distinct pathophysiological axis by combining the acute-phase protein CRP, a marker of systemic inflammation produced by hepatocytes in response to interleukin-6, with serum albumin, a negative acute-phase reactant that also reflects nutritional reserve (Gabay and Kushner, 1999; Ranzani et al., 2013). The correlation analysis in our study confirmed that CAR provides information largely independent of the cell-based markers (Spearman’s rho = −0.06 with NLR), thereby justifying its inclusion as a complementary inflammatory dimension.

An important caveat emerging from this study is the outcome-specific performance of the composite index. For poor functional outcome, IBI significantly outperformed SII (*p* = 0.012) and CAR (*p* = 0.004), but its advantage over NLR alone did not reach statistical significance (*p* = 0.226). More notably, for SAP prediction, NLR actually achieved a marginally higher AUC (0.615) than IBI (0.614), with the comparison yielding *p* = 0.641. This negative finding is biologically plausible. SAP is primarily an infectious complication driven by aspiration risk and immunosuppression, and the NLR may more directly capture the relevant pathophysiological mechanism—specifically, the combination of heightened innate immune activation and adaptive immune suppression that simultaneously promotes tissue damage and impairs antimicrobial defense (Chamorro et al., 2012; Finlayson et al., 2011). The addition of platelet dynamics (SII) and protein-level inflammation (CAR) may dilute the specific signal relevant to pneumonia susceptibility. From a clinical standpoint, these results do not support the use of IBI for SAP prediction; NLR—being simpler, cheaper, and universally available—remains the preferred biomarker when assessing pneumonia risk in PBH patients. This distinction prevents overgeneralization of IBI’s utility and highlights the fundamental principle that biomarker selection must be tailored to the specific pathophysiological mechanism of the target outcome, rather than assuming composite indices are universally superior.

The dose–response analysis using restricted cubic splines revealed a monotonically increasing relationship between IBI and poor outcome, with no evidence of a threshold effect or U-shaped pattern. This finding is clinically meaningful because it suggests that even moderate elevations in the inflammatory burden are associated with incrementally worse prognosis, rather than risk being confined to the extreme end of the spectrum. Such a graded relationship supports the use of IBI as a continuous variable for risk stratification and argues against binary cutoff-based approaches that may lose prognostic information. The absence of non-linearity also reinforces the validity of using IBI as a linear predictor in the logistic regression framework, increasing confidence in the model estimates (Harrell, 2015).

When interpreting the prognostic role of IVH in our cohort, it should be borne in mind that IVH is not a homogeneous entity; its differential diagnosis—particularly the rare non-vascular structural-related subtype—can be challenging without dedicated cerebrovascular imaging (Chen et al., 2025). With this caveat in mind, several limitations of this study warrant acknowledgment. First and foremost, the retrospective single-center design and the relative rarity of brainstem hemorrhage inherently limit the generalizability of our findings. Although our cohort of 347 patients is sizable, it was drawn from a single high-volume tertiary center, potentially introducing referral bias and restricting demographic diversity. Therefore, these findings remain hypothesis-generating; rigorous prospective external validation in independent cohorts is a prerequisite before clinical implementation can be considered. Additionally, this referral bias may partially explain our relatively low 90-day mortality rate (12.1%) compared to prior series (Murata et al., 1999; Wessels et al., 2004). Second, the IBI weighting scheme was based on a priori literature synthesis and expert judgment rather than data-driven optimization. While this approach avoids the overfitting that would occur if weights were optimized on the same dataset used for validation, it also means the weights may not be optimal for this specific population. In addition, NLR and SII were strongly correlated (rho = 0.85), so the combined 70% weight assigned to these two indices loads a largely shared leukocyte-based dimension and proportionally limits the independent contribution of CAR, which captures a distinct acute-phase and nutritional axis; data-driven or externally validated weights may therefore yield a more balanced and better-calibrated composite. Third, no correction for multiple comparisons was applied, and because several markers, subgroups, and outcomes were examined, some nominally significant associations may reflect type I error and should be regarded as exploratory. Fourth, the model’s pseudo-R^2^ of 0.084 indicates that the included predictors explain a limited proportion of the overall variance in outcomes, suggesting that important prognostic factors (such as hematoma morphology, exact brainstem location, and treatment intensity variables) were not captured. Beyond the binary presence of IVH, its topographical distribution offers additional prognostic granularity in ICH (Chen et al., 2026b). Integrating such imaging phenotyping into future PBH studies may further refine risk stratification. Fifth, the IBI components were measured at a single time point (within 24 hours of admission) and may not capture the dynamic trajectory of inflammation that evolves over the subsequent days to weeks. Serial measurements may provide additional prognostic information.

In conclusion, the composite IBI is independently associated with poor 90-day functional outcome and adds incremental discriminative value to established clinical predictors. However, as an important limitation, its utility does not extend to stroke-associated pneumonia, where the single NLR performed equally well or slightly better. This negative finding indicates that for SAP risk stratification, the simpler and more readily available NLR is clinically sufficient, and the calculation of a composite index like IBI offers no additional prognostic benefit for this specific complication. These findings collectively underscore that while multi-marker integration can enhance prognostic assessment for neurological recovery, simpler markers may suffice—or even excel—for complication-specific risk stratification. Therefore, biomarker selection should be tailored to the target outcome rather than assuming composite indices are universally superior. Future multicenter prospective studies are warranted to validate these outcome-specific associations and to refine the IBI through data-driven weighting and serial measurements before clinical translation.

## Data Availability

The raw data supporting the conclusions of this article will be made available by the authors, without undue reservation.
