# Supplementary material for: Inflammatory burden index is associated with poor functional outcome and stroke-associated pneumonia in patients with primary brainstem hemorrhage: a retrospective cohort study
Source: Front Neurosci. 2026 Sep 7;20:1873407. doi: 10.3389/fnins.2026.1873407 (PMC13590546; doi:10.3389/fnins.2026.1873407)
Supplement: Supplementary file 1 [file Supplementary_file_1.DOCX]

**Supplementary Materials**

**Supplementary Table S1. Literature sources and rationale underlying the a priori IBI weights.** The weights reflect a qualitative synthesis of published evidence and expert judgement and were not derived from the study data. The cited references concern inflammatory markers in stroke, sepsis, and oncology rather than brainstem hemorrhage specifically. An equal-weight sensitivity analysis was performed to assess robustness to the weighting choice.

| **Component** | **Assigned weight** | **Evidence basis (reference)** | **Rationale for relative weight** |
| --- | --- | --- | --- |
| NLR | 0.50 | Lattanzi 2019 [8]; Tao 2017 [9]; Giede-Jeppe 2017 [18] | Most extensive and consistent prognostic evidence in ischemic and hemorrhagic stroke. |
| CAR | 0.30 | Ranzani 2013 [11]; Gabay 1999 [22] | Established acute-phase/nutritional marker; smaller but consistent stroke evidence base. |
| SII | 0.20 | Hu 2014 [10]; Semple 2011 [21] | Prognostic value demonstrated mainly outside brainstem hemorrhage; least disease-specific evidence. |

**Supplementary Table S2. Discrimination of IBI and individual inflammatory markers for poor 90-day functional outcome and stroke-associated pneumonia.** All ROC analyses were computed within a single analytic pipeline. AUCs were compared using the DeLong test.

**(A) Discrimination for poor 90-day functional outcome.**

| Marker | AUC | 95% CI | DeLong p vs IBI |
| --- | --- | --- | --- |
| IBI | 0.663 | 0.599–0.720 | — (reference) |
| NLR | 0.638 | 0.569–0.698 | 0.226 |
| SII | 0.600 | 0.528–0.666 | 0.012 |
| CAR | 0.550 | 0.485–0.616 | 0.004 |

**(B) Discrimination for stroke-associated pneumonia.**

| Marker | AUC | 95% CI | DeLong p vs IBI |
| --- | --- | --- | --- |
| NLR | 0.615 | 0.546–0.703 | 0.641 |
| IBI | 0.614 | 0.535–0.694 | — (reference) |
| SII | 0.593 | 0.521–0.662 | 0.318 |
| CAR | 0.516 | 0.447–0.586 | 0.088 |

**Supplementary Table S3. Incremental value of IBI beyond the reference clinical model (poor 90-day outcome).**

| Model | AUC (poor outcome) | 95% CI | Metric vs reference |
| --- | --- | --- | --- |
| Reference clinical model (age, GCS, hematoma volume, IVH) | 0.623 | 0.556–0.690 | — |
| Reference model + IBI | 0.701 | 0.639–0.763 | DeLong p = 0.008 |
| Continuous NRI |  |  | 0.31 (0.10–0.52), p = 0.004 |
| IDI |  |  | 0.045 (0.021–0.069), p < 0.001 |

**Supplementary Table S4. Number of patients in each modified Rankin Scale (mRS) category by IBI tertile.** Counts share the single source used for Table 1 and Figure 4: the mRS 0–2 total equals the complement of the poor-outcome rate in Table 1 (18.1%, 27.8%, 44.8% for T1–T3) and the mRS 6 count equals the tertile-specific 90-day mortality (8.6%, 13.0%, 14.7%).

| **IBI tertile** | **mRS 0** | **mRS 1** | **mRS 2** | **mRS 3** | **mRS 4** | **mRS 5** | **mRS 6** | **mRS 0–2, n (%)** | **mRS 3–6, n (%)** |
| --- | --- | --- | --- | --- | --- | --- | --- | --- | --- |
| T1 (Low, n=116) | 30 | 40 | 25 | 4 | 4 | 3 | 10 | 95 (81.9) | 21 (18.1) |
| T2 (Medium, n=115) | 25 | 35 | 23 | 7 | 6 | 4 | 15 | 83 (72.2) | 32 (27.8) |
| T3 (High, n=116) | 15 | 28 | 21 | 14 | 13 | 8 | 17 | 64 (55.2) | 52 (44.8) |
| Total (n=347) | 70 | 103 | 69 | 25 | 23 | 15 | 42 | 242 (69.7) | 105 (30.3) |

**Supplementary Table S5. Sensitivity analyses for the association between IBI and poor 90-day functional outcome.**

The primary IBI used a priori weights (0.50×NLRz + 0.30×CARz + 0.20×SIIz). To assess robustness to the weighting choice, the composite was recomputed with equal weights (one-third each) and re-entered into the multivariable model adjusted for age, GCS, hematoma volume, and IVH. The direction, magnitude, and statistical significance of the association were unchanged, indicating that the findings were not driven by the specific a priori weights.

| **Analysis (multivariable model)** | **IBI weighting** | **IBI OR (95% CI)** | **p** |
| --- | --- | --- | --- |
| Primary analysis | a priori (0.50/0.30/0.20) | 2.31 (1.64–3.26) | <0.001 |
| Sensitivity analysis | equal (1/3, 1/3, 1/3) | 2.24 (1.59–3.16) | <0.001 |

***Notes:*** *Both models were adjusted for age, GCS, hematoma volume, and IVH. OR, odds ratio; CI, confidence interval; IBI, Inflammatory Burden Index; NLR, neutrophil-to-lymphocyte ratio; CAR, C-reactive protein-to-albumin ratio; SII, systemic immune-inflammation index.*
